# Evaluation of severe adverse events during rehabilitation for acute-phase patients: A retrospective cohort study

**DOI:** 10.1097/MD.0000000000029516

**Published:** 2022-06-24

**Authors:** Tokio Kinoshita, Yoshi-Ichiro Kamijo, Ken Kouda, Yoshinori Yasuoka, Yukihide Nishimura, Yasunori Umemoto, Takahiro Ogawa, Yukio Mikami, Makoto Kawanishi, Fumihiro Tajima

**Affiliations:** a Department of Rehabilitation Medicine, Wakayama Medical University, 811-1 Kimiidera, Wakayama, Wakayama, Japan; b Division of Rehabilitation, Wakayama Medical University Hospital, 811-1 Kimiidera, Wakayama, Wakayama, Japan; c Department of Rehabilitation Medicine, Dokkyo Medical University Saitama Medical Center, 2-1-50 Minami-Koshigaya, Koshigaya city, Saitama, Japan; d Department of Rehabilitation Medicine, Iwate Medical University, 2-1-1 Idaidouri, Yahaba-cho, Shiwa-gun, Iwate, Japan; e Chuzan Hospital Clinical Education and Research Center, 6-2-1 Matsumoto, Okinawa, Okinawa, Japan.

**Keywords:** administration in rehabilitation, inpatient rehabilitation, quality improvement and patient safety

## Abstract

Early mobilization decreases the likelihood of negative outcomes for acute-phase inpatients. Adverse events occurring during intensive care unit rehabilitation have previously been reported; however, no study has reported the incidence rates for adverse events during the acute rehabilitation phase. This study aimed to investigate the incidence of severe adverse events during acute-phase rehabilitation and evaluate them in detail.

Reports of adverse events occurring during acute-phase rehabilitation in a university hospital from April 1, 2011 to March 31, 2018 were retrospectively assessed.

Nine severe adverse events occurred during this period (incidence rate, 0.032%), comprising 2 cardiopulmonary arrests, 2 pulseless electrical activity events, 2 deterioration in consciousness events, 1 deterioration in consciousness event due to cerebral infarction, 1 fracture due to a fall, and 1 event involving removal of a ventricular drain. Pulmonary thromboembolism was implicated in 1 adverse event involving pulseless electrical activity and 1 deterioration in consciousness event. The causes for the 6 other adverse events could not be identified. The mean days from admission and the onset of rehabilitation to adverse event occurrence were 22.0 ± 18.2 and 17.9 ± 13.5 days (mean ± standard deviation), respectively. Four of 9 patients died, and 5 patients were discharged home or transferred to other stepdown facilities. When assessed retrospectively, there were no conflicts between patient conditions and the cancellation criteria of rehabilitation by the Japanese Association of Rehabilitation Medicine.

The occurrences of severe adverse event may not be related to early mobilization (or onset time of rehabilitation) and compliance status of cancellation criteria.

## 1 Introduction

The effects of early mobilization from intensive care units (ICUs) on inpatients has been previously demonstrated.^[1–4]^ Furthermore, the effects of early mobilization on patients with stroke has also been proven,^[5,6]^ and many guidelines strongly recommend early mobilization.^[7–9]^ However, it may possibly increase the risk of adverse events if diagnosis and symptom management are inappropriate. In the United States, medical malpractice in hospitals is a serious issue,^[10–12]^ with one or more adverse events (AEs) due to hospital care having been reported to occur in 3% to 17% of inpatients in acute-phase hospitals in the United States and Europe, regardless of rehabilitation.^[13]^ Risk management strategies for early mobilization should be considered.

Severe adverse events (SAEs) may prolong a hospital stay, and may have medical sequelae along with the possibility of litigation, resulting in increasing medical cost burdens. Two studies reported that adverse events occurring during rehabilitation in ICUs mostly involved no additional treatment, cost, and/or extension of hospitalization.^[14,15]^ However, these studies did not specify the target patient populations, nor were the medical safety measures described, and these results should be interpreted with caution. No studies concerning inpatients in acute-phase hospitals have reported SAEs during early mobilization.

Our medical hospital is an acute care and tertiary emergency medical facility. Rehabilitation therapies are provided by a specialized team comprised of physiatrists and registered therapists operating rehabilitation, depending on the condition of each patient under medical management.^[5,6]^ At our hospital, we have experienced several SAEs during rehabilitation. In this study, we retrospectively assessed our department's reports of SAEs. This study aimed to evaluate the SAE incidence rate and obtain detailed information for future improvement to our risk management strategy.

## 2 Methods

### 2.1 Study setting

Our hospital has 760 general beds (including ten ICU beds) and 40 psychiatric beds, serving 27 clinical departments and 22 central medical treatment sections. Physiatrists examine inpatients prior to starting rehabilitation and evaluate their diagnosis, disease state, and physical condition. Registered and skilled therapists then commence exercise therapy. A team meeting is held in the evening of the patient referral day to identify challenges and consider solutions, with the aim of improving rehabilitation safety and efficacy. Patient status is assessed every morning for all patients participating in rehabilitation sessions. Therapists measure vital signs pre- and postrehabilitation. Rehabilitation therapies are performed based on a thorough clinical examination and in accordance with each patient's condition.^[5,6]^

### 2.2 Study design

In this retrospective cohort study, we analyzed SAE reports that had been submitted to the Medical Safety Promotion Department at our hospital from the Department of Rehabilitation Medicine between April 2011 and March 2018, during which time 1 author (T.K.) worked as the department's patient safety manager.

### 2.3 Data collection methods and procedures

At our hospital, all staff are required to report an AE to their relevant risk manager each time an AE occurs. A decision is made concerning the AE level by the Medical Safety Promotion Department at our hospital. According to the National Coordination Council for Medication Error Reporting and Prevention index, AEs are categorized into nine levels, as follows^[16]^: Category A (no error); categories B to D (error but no harm); categories E to H (error and harm), and category I (error and death). For adverse events that occur in categories A to D, no additional treatment is required. For adverse events that occur in category E, minor treatment is required. For adverse events that occur in category F, intensive treatments are required and/or extension of hospital stay is needed. If permanent disability and sequelae with no significant or with significant functional or cosmetic problems develop, SAEs are defined as G or H, respectively; thus, SAEs are defined as categories F to I in reference to the above index.

Rehabilitation period was defined as the period when a therapist provided rehabilitation in a patient's bedroom, corridor, training room (or outdoors). Specific definitions of the period include durations

1. from when a therapist enters patient's room until leaving,
2. from when a patient enters a training room until leaving, or
3. if a surveillance and/or assistance patient was moved with a transfer staff, duration was defined as from when a therapist received a patient to when a patient was passed over to a transfer staff member.

We investigated the numbers of hospitalized patients, patients (inpatients and outpatients) referred to our department, deaths, discharge, length of admission, days until the onset of rehabilitation, and total numbers of AEs, namely, non-SAEs or SAEs that occurred in our hospital during the study period, and the total number of patients who underwent rehabilitation annually during the study period.

Data concerning background factors (age, sex, height, weight), the department that had mainly treated the patients with SAEs, the primary disease, and comorbidities were obtained from the SAE reports. We also obtained data concerning contents and causes, the place of SAE occurrence, occurrence status, whether a stat call was made, the number of days from admission to starting rehabilitation to SAE occurrence and to discharge, days from surgery to SAE occurrence, discharge status (home discharge, transfer to a stepdown facility, death discharge), predictability determined by the Medical Safety Promotion Department, and therapist experience years. For all patients with SAEs, we retrospectively verified whether a patient's status conformed with Guidelines for Safety Management and Promotion cancellation criteria for rehabilitation, which had been edited by the Clinical Practice Guidelines Committee of the Japanese Association of Rehabilitation Medicine in Japan^[17]^ (Table 1).

**Table 1:** Criteria for cancellation and rehabilitation cessation reported by the Clinical Guideline Committee of the Japanese Association of Rehabilitation Medicine.

Conditions where strenuous rehabilitation should be avoided
Resting pulse rate ≤40 or ≥120 beats min^−1^
Resting systolic blood pressure ≤70 or ≥200 mm Hg
Resting diastolic blood pressure ≥120 mm Hg
Shortness of breath before rehabilitation
Exertional angina pectoris
Marked bradycardia or tachycardia in patients with atrial fibrillation
Low cardiovascular hemodynamics in patients soon after myocardial infarction
Marked arrhythmia
Resting chest pain
Palpitations, or shortness of breath, or chest pain before rehabilitation
Dizziness, cold perspiration, nausea, etc. in seated position
Resting body temperature > 38°C
Resting oxygen saturation ≤90%
Conditions where rehabilitation should be cancelled
Higher than moderate degree of shortness of breath, dizziness, nausea, angina pectoris, headache, a strong feeling of fatigue, etc.
Pulse rate > 140 beats min^−1^
Systolic and diastolic blood pressures elevated > 40 or > 20 mmHg during exercise, respectively
Tachypnea (>30 breaths min^−1^) or shortness of breath
Increase in arrhythmia during exercise
Bradycardia
Worsening consciousness
Conditions where rehabilitation should be stopped and may be resumed after confirmation of recovery
Pulse rate exceeds 30% before exercise: cancel or change to very light exercise if it does not return to less than 10% before exercise after 2-min rest
Pulse rate >120 beats min^−1^
≥10 premature ventricular contractions in 1 min
Mild palpitation or shortness of breath
Conditions where other precautions are required
Hematuria
Increase in sputum
Increase in body weight
Fatigue
Appetite loss or starving
Worsening edema of the lower extremity

This table was modified by the authors based on reference [17] (in Japanese).

### 2.4 Statistical analyses

The values of the variables are given as numbers, %, mean ± standard deviation (SD), or median (min-max) where applicable.

### 2.5 Ethical considerations

This study was conducted in accordance with the Declaration of Helsinki and its protocol was approved by the relevant ethics review committee (No. 2677). In this retrospective study, there were no additional risks to patients during the data collection and analysis. All information related to the patients was protected. Information concerning this study was posted on the university website and patients or their families and relatives were given the opportunity to opt-out. The ethics review committee waived the requirement for written informed patient consent due to the study's retrospective design.

## 3 Results

The average annual number of inpatients during the study period was 16231.9 ± 646.4, and the average length of admission was 14.6 ± 0.6 days (Table 2). All departments, except the Department of Anesthesia, referred patients to our department for rehabilitation, which included: Emergency and Critical Care Medicine (19.0%), Orthopedic Surgery (14.2%), Gastroenterological Surgery (12.4%), Hematology and/or Oncology (6.7%), Neurosurgery (6.3%), Cardiovascular and/or Respiratory Surgery (5.8%), Cardiovascular Medicine (5.6%), and Neurology (4.5%) up to the last year of the study period. The annual number of new referrals was 2839 (18.4%, referrals/total inpatients ratio) in the first year, which increased to 4759 referrals in the last year of the study period (27.9%). The average number of days from admission to rehabilitation referrals was 4.1 ± 7.6 days in the last year of the study period. The number of inpatients provided with rehabilitation was approximately 310 per day.

**Table 2:** Inpatient descriptive statistics and referrals to the rehabilitation medicine department every year during the study period at our hospital.

	2011.4 to 2012.3	2012.4 to 2013.3	2013.4 to 2014.3	2014.4 to 2015.3	2015.4 to 2016.3	2016.4 to 2017.3	2017.4 to 2018.3	Total numbers	Average ± SD
Number of hospitalized patients	15013	15786	16091	16517	16636	16522	17058	113623	16231.9 ± 646.4
Average length of stay (day)	15.7	15.0	14.5	14.1	14.1	14.5	14.3		14.6 ± 0.6
Number of patients referred to the rehabilitation departments	3125	3233	3413	4039	4295	4673	5189	27967	3995.3 ± 749.7
Inpatients	2839	2899	3008	3635	3956	4291	4759	25387	3626.7 ± 719.5
Outpatients	286	334	405	404	339	382	430	2580	368.6 ± 48.7
Referral rate to the rehabilitation department (inpatient referrals / total hospitalizations)	18.9%	18.4%	18.7%	22.0%	23.8%	26.0%	27.9%		22.3 ± 3.7%
Days until start of rehabilitation Average ± SD Data loss rate	No data 100%	No data 100%	6.8 ± 17.94.3%	5.8 ± 12.220.3%	5.8 ± 12.811.9%	5.3 ± 12.410.3%	4.1 ± 7.60.1%		

SD = standard deviation.

Table 3 lists the nine SAEs reported during rehabilitation over the seven-year study period, with an annual occurrence of 0 to 3 SAEs per year.

**Table 3:** Numbers of inpatient adverse events.

	2011.4 to 2012.3	2012.4 to 2013.3	2013.4 to 2014.3	2014.4 to 2015.3	2015.4 to 2016.3	2016.4 to 2017.3	2017.4 to 2018.3	Total numbers	Average ± SD
Number of adverse events during rehabilitation	141	138	166	159	112	133	110	959	137.0 ± 20.4
Number of non-severe adverse events	140	138	163	158	111	132	108	950	135.7 ± 20.2
Number of severe adverse events	1	0	3	1	1	1	2	9	1.3 ± 0.9

SD = standard deviation. Non-severe adverse events were defined as categories A to E, and severe adverse events were defined as categories F to I according to the National Coordinating Council for Medication Error Reporting and Prevention.

Table 4 summarizes all nine SAEs that had occurred during rehabilitation. The average patient age was 77.7 ± 7.2 years (5 males, 4 females), and patients had been referred from the following primary departments: Neurosurgery (n = 3), Orthopedic Surgery (n = 2), Cardiovascular Medicine (n = 1), Gastroenterological Surgery (n = 1), Cardiovascular Surgery (n = 1), and Emergency and Critical Care Medicine (n = 1). There were 2 pulseless electrical activity SAEs of unknown causes (Cases A and E). The SAE in Case A occurred due to a pulmonary thromboembolism of undetermined origin on day 1 at the onset of walking training, and the SAE in Case E occurred due to unknown causes on day 4 at the onset of walking training. Two cardiopulmonary arrests due to unknown causes were reported for Cases C and G. A deterioration in consciousness and vital signs of unknown cause was reported for Case B (with no details concerning the examination or treatment reported due to a Do Not Attempt Resuscitation order) and, in Case D, due to a pulmonary thromboembolism of undetermined origin. Loss of consciousness, dysarthria, and hemiplegia occurred due to cerebral infarction in Case H. A femoral fracture occurred due to a fall in Case I. In Case F, a ventricular drain was removed by a therapist. Eight patients had SAEs that occurred during walking and/or standing training and, in 1 case (Case E), an SAE occurred just after arrival at the training room. Of these patients, 4 died in hospital, 3 were discharged home, and 2 were transferred to other stepdown facilities. The Medical Safety Promotion Department deemed no patients had died because of rehabilitation. Time from admission to the onset of rehabilitation was 4.1 ± 5.4 days (0–18 [min-max]). The period between hospital admission and SAE occurrence was 22.0 ± 18.2 (range, 7–69) days. The period between hospital admission and discharge was 53.7 ± 39.2 (range, 18–119) days. The number of days from the start of rehabilitation to SAE occurrence was 17.9 ± 13.5 (range, 5–51) days. At the time of SAE occurrence, the training program had been conducted for 8.9 ± 7.6 (1–20) days. The responsible therapist had an average of 8.1 ± 6.9 years of experience. Rehabilitation therapies were provided in accordance with cancellation criteria in all patients, except for Case F that involved an SAE that occurred just upon arrival at the rehabilitation room before the clinical examination.

**Table 4:** Severe adverse event case summary.

	Case A	Case B	Case C	Case D	Case E	Case F	Case G	Case H	Case I	Average ± SD
Age (yr)	68	88	84	71	75	68	84	82	79	77.7 ± 7.2
Sex	Female	Female	Male	Female	Female	Male	Male	Male	Male	
Height (cm)	155	142	No data	150.3	146	163.1	153	166.9	165.3	15.2 ± 8.9
Body weight (kg)	77.0	52.7	No data	50.7	47.0	56.1	43.6	70.2	71.9	58.7 ± 12.2
Primary department	Ortho Surg.	Cardio Med.	Neuro Surg.	Gastro Surg.	Neuro Surg.	Neuro Surg.	Emerg	Cardio Surg.	Ortho Surg.	
Primary disease	Pyogenic spondylitis	Large atrial thrombus, AS, MS	Subcortical hemorrhage	Postop. of duodenal cancer	Cardiogenic cerebral infarction	Thalamic hemorrhage	Sepsis, pyothorax, postop. of ileus	Postop. of cardiac myxoma, angina (Tumor removal, CABG)	Post TKA	
Comorbidities	DM	Kidney injury (HD), Af, HT, DL	Dementia	Knee OA	HT, Af, Paf, DM, HU, DL	DM, HT	HT, DM, RA, interstitial pneumonia	Cerebral infarction, Af, DM, DL	HT, HU	
Contents of severe adverse events	PEA due to PTE	Loss of conscious-ness and vital signs due to unknown causes (DNAR)	CPA due to unknown origin	Loss of conscious-ness and vital signs due to PTE	PEA due to unknown	Ventricular drain removal	CPA due to unknown cause	Loss of consciousness, dysarthria, and hemiplegia due to Cerebral infarction	Femur fracture due to a fall	
Place at occurrence	Training room	Corridor around the training room	Training room	Ward corridor	On arrival in the training room	Patient's bedroom	Training room	Training room	Corridor near the training room	
Training situation at the occurrence	1st day of walking	17th day of gait	17th day of gait	2nd day of gait	4th day of walking	3rd day of being upright	12th day of being upright	4th day of gait	20th day of gait	8.9 ± 7.6
Stat call request	did	none	did	none	did	none	did	none	none	
Days from admission to rehabilitation onset to occurrence	1869	320	116	09	618	310	426	27	023	4.1 ± 5.422.0 ± 18.2
Days until events occurrence from rehabilitation from surgery	51N/A	17N/A	15N/A	92	12N/A	710	2226	56	2321	17.9 ± 13.5N/A
Days until discharge from admission from occurrence	723	200	118102	3728	180	3727	4418	1811	11996	53.7 ± 39.231.7 ± 38.4
Discharge status	Death	Death	Trans.	Home	Death	Trans.	Death	Home	Home	
Therapist experience years	5	6	1	22	6	2	7	18	6	8.1 ± 6.9
Adherence status of the cancellation criteria	Within	Within	Within	Within	N/A	Within	Within	Within	Within	

Af = atrial fibrillation, AS = aortic stenosis, CABG = coronary artery bypass grafting, Cardio Med. = cardiovascular medicine (internal medicine), Cardio Surg. = cardiovascular surgery, CPA = cardiopulmonary arrest, DL = dyslipidemia, DM = diabetes mellitus, DNAR = do not attempt resuscitation, Emerg. = emergency and critical care medicine, Gastro Surg. = gastroenterological surgery, HD = hemodialysis, HT = hypertension, HU = hyperuricemia, MS = mitral stenosis, N/A = not applicable, Neuro Surg. = neurological surgery, OA = osteoarthritis, Ortho Surg. = orthopedic surgery, Paf = paroxysmal atrial fibrillation, PEA = pulseless electrical activity, Postop. = postoperative, PTE = pulmonary thromboembolism, RA = rheumatoid arthritis, TKA = total knee arthroplasty, Trans. = transferred to other stepdown facilities Values are shown as means.

## 4 Discussion

This study is the first to evaluate SAEs occurring during acute-phase rehabilitation. At our hospital, a specialized team of rehabilitation physiatrists and registered therapists provide rehabilitation^[5,6]^ based on daily morning patient assessments of disease status and physical condition, along with a team meeting every evening. The incidence rate of SAEs during rehabilitation was 0.032% (9 events in 27,967 rehabilitation patients × 100) for the seven-year period. Two cardiopulmonary arrests and 2 pulseless electrical activities were involved.

Previous studies have reported that, in patients with stroke and in patients in ICU, early rehabilitation intervention did not increase the number of deaths, which supports our results.^[5,6,18,19]^ In our study, the period from admission (18 days; 5–51 [min–max]) or from the onset of rehabilitation to the occurrence of an SAE (22 days; 9–69 [min–max]) was approximately 20 days, which indicated that early onset of rehabilitation was unlikely to be related to SAE occurrence. Furthermore, only in Case A did an SAE occur on the day when the exercise load was increased. In the other 8 cases, the rehabilitation program at the time of SAE occurrence had begun being performed from at least the day before (10 days; 2–20 [min– max]). Therefore, it appears unlikely that SAE occurrences were related to increased exercise loads.

A previous report investigating the occurrence of AEs during rehabilitation among acute-phase hospital, rehabilitation hospital, and nursing home inpatients reported a negative correlation between SAEs occurrences and therapist years of experience.^[20]^ However, as the years of experience among the responsible therapist in charge of each patient was approximately 8 years (1–22 years) in the 9 cases presented in this study, with considerable variation, it would appear that the number of years of experience was not associated with the occurrences of SAEs.

All the SAEs in this study accorded with established cancellation criteria, which mainly comprise an evaluation of vital signs and chest symptoms used in early detection of status changes in patients. There is a need to be aware of the risk of thrombus formation during rehabilitation, especially in an acute phase. In a previous study of 11,786,489 surgeries from 6530 hospitals during a ten-year period from 2002 to 2012, pulmonary thromboembolism was reported in 3667 perioperative patients with an incidence rate of 0.031%.^[21]^ Furthermore, 57% of acute pulmonary thromboembolic events have been reported to occur during standing or walking^[22]^ and 22% to 53% have occurred during defecation or urination.^[23]^ Most emboli were derived from venous thrombi in the lower limbs and pelvis, and muscle contraction of the lower limbs when standing, walking, or during defecation increases venous return to the heart due to the action of the muscle pump, at which time a thrombus can be released and an acute pulmonary thromboembolism may occur.^[24]^ However, 1 meta-analysis reported that early gait training was not associated with an increased incidence of new pulmonary thromboembolisms, deep vein thrombosis (DVT) progression, or DVT-related mortality when compared with patients not undertaking gait training.^[25,26]^ Moreover, Japanese guidelines recommend early walking for patients with DVT.^[24]^

Of the 2 episodes of pulmonary thromboembolism in our study, 1 patient had started to get out of bed more than 1 month earlier and had continued standing training and started walking training prior to the day of the SAE (Case A). In another case, rehabilitation had been performed preoperatively, and walking training recommenced the day after surgery, with the pulmonary thromboembolism occurring when the walking distance was extended from the second postoperative day (Case D). Rehabilitation medical care may not increase the risk of the occurrence of pulmonary thromboembolism; however, it is necessary to prepare for its possible occurrence at any time during rehabilitation.

This study was limited in that it was a single-center, retrospective cohort study; therefore, information concerning a non-adverse event group was not available. Furthermore, due to the small sample size, statistical analyses other than in relation to descriptive statistics were not possible.

## 5 Conclusion

Nine SAEs occurred during acute-phase rehabilitation over a seven-year study period, and rehabilitation did not increase the occurrence of SAEs in our hospital. SAEs were not found to be related to early mobilization (or onset time of rehabilitation), an increased exercise load, or therapists’ years of experience. In clinical acute rehabilitation medicine, SAEs that require intensive care might occur regardless of whether they accord with Japanese Association of Rehabilitation Medicine cancellation criteria. Rehabilitation should be performed depending on each patient's status under medical management as undertaken by physiatrists.

## Acknowledgments

The authors thank Mrs. Yuta Minoshima, Yuri Matsuura, Daichi Shima, Shinji Kawasaki, and Shinnosuke Hori for their excellent assistance with data collection. We also thank Dr. Sven P. Hoekstra for kindly editing of the manuscript. This work was supported by the Nachikatsuura Research Foundation [grant numbers 1221].

## Author contributions

**Conceptualization:** Tokio Kinoshita, Yoshi-Ichiro Kamijo, Yukihide Nishimura.

**Data curation:** Makoto Kawanishi, Takahiro Ogawa, Tokio Kinoshita, Yasunori Umemoto, Yoshinori Yasuoka.

**Formal analysis:** Makoto Kawanishi, Yoshinori Yasuoka.

**Investigation:** Yoshi-Ichiro Kamijo.

**Methodology:** Takahiro Ogawa, Yoshi-Ichiro Kamijo.

**Project administration:** Fumihiro Tajima.

**Supervision:** Fumihiro Tajima, Yukihide Nishimura.

**Validation:** Ken Kouda.

**Writing – original draft:** Tokio Kinoshita, Yoshi-Ichiro Kamijo.

**Writing – review & editing:** Fumihiro Tajima, Ken Kouda, Yasunori Umemoto, Yoshi-Ichiro Kamijo, Yukihide Nishimura, Yukio Mikami.
